# Alcohol-related risk perception in primary care patients screening positive for unhealthy alcohol consumption

**DOI:** 10.1186/1940-0640-10-S2-O9

**Published:** 2015-09-24

**Authors:** Gallus Bischof, Anja Bischof, Christian Meyer, Hans-Jueegen Rumpf

**Affiliations:** 1 Dpt. of Psychiatry, University of Luebeck, Luebeck, Germany; 2 Institute of Epidemiology and Social Medicine, University of Greifswald, Greifswald, Germany

## Background

Among unhealthy drinking patterns, drinking above recommended limits is the most prevalent one. Little is known to what extend lack of knowledge on risky amounts of drinking influences these drinking patterns.

## Material and methods

In general practices in Luebeck, Germany, all consecutive patients aged 18-64 were systematically screened. Individuals screening positive on the Alcohol Use disorders Identification test (AUDIT) or the Luebeck Alcoholism Screening Test (LAST) were asked to participate in an intervention study. Patients screening positive willing to participate (n=801; response rate: 54.3%) were diagnosed using the Munich Composite International Diagnostic Inventory M-CIDI. In addition to Alcohol Use Disorders (AUDs), at-risk drinking according to the British Medical Association (20/30 g/alc per day females/males) and binge drinking (50/60 g/alc in one sitting females/males) were assessed. As a part of an in-depth assessment, participants were asked to rate how many standard drinks one could drink on a daily basis without having to fear negative consequences. In the present analysis, participants were dichotomized according to BMA-recommendations into individuals with gender-specific adequate (ARP, n= 684) and individuals with inadequate (INARP, n=75) risk perception. These groups were compared.

## Results

Patients with INARP had a lower degree of schooling, were more often male and reported more often at-risk drinking according to BMA but did not report higher levels of binge drinking or AUDs.

## Conclusions

Data suggest that in a subgroup of at-risk drinkers, lack of knowledge concerning health-related drinking limits might perpetuate unhealthy drinking patterns. This might in part also explain findings on the beneficial impact of minimal interventions (e.g. simple advice) on unhealthy drinking patterns.

